# Mesenchymal stromal cell therapy for COVID-19-induced ARDS patients: a successful phase 1, control-placebo group, clinical trial

**DOI:** 10.1186/s13287-022-02920-1

**Published:** 2022-06-28

**Authors:** Najmeh Kaffash Farkhad, Alireza Sedaghat, Hamidreza Reihani, Amir Adhami Moghadam, Ahmad Bagheri Moghadam, Nayereh Khadem Ghaebi, Mohammad Ali Khodadoust, Rashin Ganjali, Amir Reza Tafreshian, Jalil Tavakol-Afshari

**Affiliations:** 1 Department of Immunology, Immunology Research Center, Faculty of Medicine, Mashhad University of Medical Sciences, Mashhad, Iran; 2 Student Research Committee, Faculty of Medicine, Mashhad University of Medical Sciences, Mashhad, Iran; 3 Lung Disease Research Center, Faculty of Medicine, Mashhad University of Medical Sciences, Mashhad, Iran; 4 Department of Emergency Medicine, Faculty of Medicine, Mashhad University of Medical Sciences, Mashhad, Iran; 5 Department of Internal Medicine and Critical Care, Islamic Azad University, Mashhad Branch, Mashhad, Iran; 6 Specialty of Internal Medicine and Critical Care, Head of Army Hospital ICU and Intensive, Mashhad, Iran; 7 Department of Anesthesiology, Faculty of Medicine, Mashhad University of Medical Sciences, Mashhad, Iran; 8 Neonatal Research Center, Faculty of Medicine, Mashhad University of Medical Sciences, Mashhad, Iran

**Keywords:** Mesenchymal stromal cells, Safety, Clinical trial, Acute respiratory distress syndrome

## Abstract

**Background:**

Acute respiratory distress syndrome (ARDS) is the devastating complication of the new COVID-19 pandemic, directly correlated with releasing large amounts of inflammatory cytokines. Due to their immunoregulatory features, mesenchymal stromal cells (MSCs) provide a promising approach against this disease. In this regard, this study was designed as a single-center, open-label, phase 1 clinical trial with a control group to examine the safety and explore the possible potency of three injections of umbilical cord-derived MSCs (UC-MSCs) in mild–moderate COVID-19-induced ARDS patients.

**Methods:**

Twenty confirmed COVID-19 patients with mild-to-moderate ARDS degree entered the study and were divided into two groups: control group (standard care) and intervention group (standard care + UC-MSCs). The patients received three intravenous infusions of UC-MSCs (1 × ${10}^{6}$ cells/kg BW per injection) every other day. Respiratory markers, CRP levels and specific serum cytokines were assessed four times (days of 0, 5, 10 and 17) during the 17-day follow-up period.

**Results:**

During the study, there were no serious adverse effects after cell transplantations. Besides, significant improvement in SPO_2_/FIO_2_ ratio and serum CRP levels was observed. On the other hand, a significant decrease (*P* < 0.05) in serum cytokine levels of IL-6, IFN-g, TNF-α, IL-17 A and a significant increase in serum cytokine levels of TGF-B, IL-1B and IL-10 were observed. Also, no significant changes were observed in CT scan images of patients during the study period.

**Conclusion:**

Our obtained results demonstrated that multiple intravenous transplantations of allogenic UC-MSCs in non-severe COVID-19-induced ARDS patients are a safe procedure. In addition, this intervention is a hopeful approach to decline cytokine storm and recover respiratory functions. Indeed, more clinical trials with larger sample sizes are required to confirm these results.

*Trial registration* This clinical trial was registered with the Iranian Registry of Clinical Trials (ID: IRCT20160809029275N1 at 2020.05.30).

## Introduction

COVID-19, a viral infectious disease caused by a new coronavirus (SARS-CoV-2), first appeared in December 2019 in China [1, 2] and quickly became a global pandemic [3, 4]. The lower respiratory tract, especially the lungs, is the key targets [5] which are easily accessible by the virus via angiotensin-converting enzyme 2 (ACE2) receptor expressed on pulmonary epithelial cells [6]. Through the abundance of ACE2 on different organs like the kidney, nervous system, lungs, liver and heart, multi-organ failure is a relatively common complexity of these patients when the disease progresses severely [6]. Acute respiratory distress syndrome (ARDS), the devastating characteristic complexity of COVID-19 disease with high mortality [7], is caused by different mechanisms, including overactivation of neutrophils, the release of a large number of inflammatory cytokines (cytokine storm) and the renin–angiotensin system dysregulation [8]. ARDS is a generalized destructive lung injury characterized by pulmonary edema and endothelial damage that leads to progressive respiratory failure [9]. It has been proven that ARDS is a common and crucial reason for the intensive care unit (ICU) admission of COVID-19 patients [10, 11]. In this regard, applying therapeutic methods to reduce the inflammation and improve the lung's status can be a useful treatment strategy for COVID-19-induced ARDS patients. MSCs are considered suitable therapeutic candidates in this field due to their reparative, anti-inflammatory, immunomodulatory and migratory properties that stimulate tissue repair and interact inflammation [12].

MSCs are non-hematopoietic, qualified cells characterized by self-renewal ability and potential of differentiating into multiple cell lines [13]. So far, they have effectively been used in several studies for treatment of numerous disorders, including systemic lupus erythematosus (SLE) [14], amyotrophic lateral sclerosis (ALS) [15], graft-versus-host disease (GVHD) [16] and ARDS [17–20]. In this regard, many researches have also been registered on the use of these cells for COVID-19 patients (https://clinicaltrials.gov), which only a few of them have been completed and published their results [21, 22]. It has been revealed that MSCs are safe [23], ACE2 negative [7] and can effectively inhibit the over-activated immune system in COVID-19 patients [24]. Also, the intravenous (IV) injection of MSCs can rapidly carry a high number of them to the lungs [25] as the main injured organs in ARDS [26]. Based on these trials and available information, in this study we aim to evaluate the safety of 3 doses injection of UC-MSCs in COVID-19-induced ARDS patients. Also, it is an exploratory pilot study to determine possible changes in specific biomarkers of inflammatory dysregulation and their alteration following MSCs therapy in these patients.

## Materials and methods

### Trial design

This study was a single-center, open-label, phase 1 clinical trial with a placebo-control group conducted at Imam Reza Hospital, Mashhad University of Medical Sciences, Mashhad, Iran. It was performed on 20 COVID-19-induced ARDS patients following the Declaration of Helsinki Ethical Principles and approved by the Ethics Committee of the Mashhad University of Medical Sciences, Mashhad, Iran (IR. MUMS. REC.1399.150). Also, this clinical trial was registered with the Iranian Registry of Clinical Trials (ID: IRCT20160809029275N1 at 2020.05.30).

### Product preparation and characterization

**Fig. 1:**
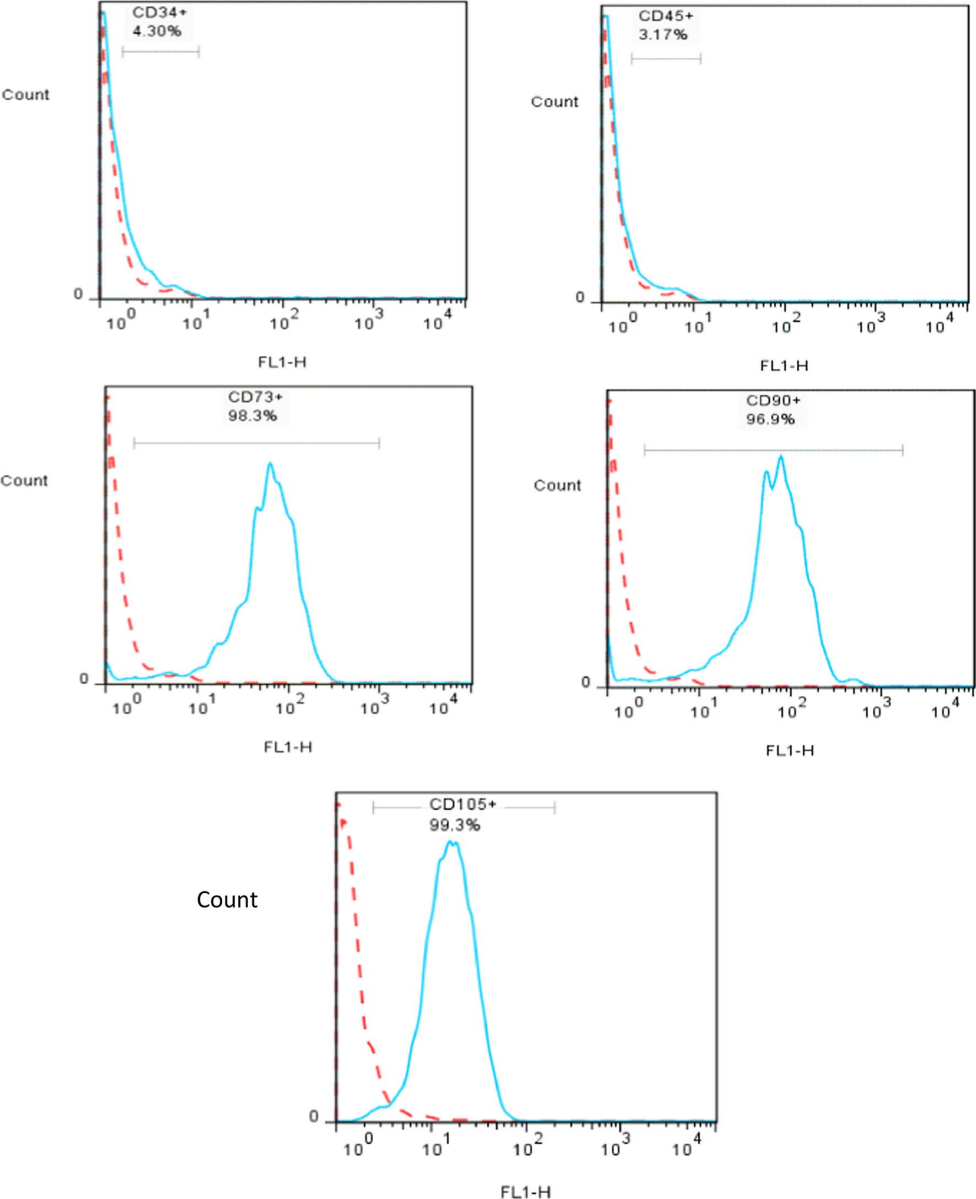
Immunophenotypic characterization of human umbilical cord-derived mesenchymal stromal cells by flow cytometry for the expression of mesenchymal (CD73, CD90, CD105) and hematopoietic (CD34, CD 45) stem cells markers. (Dotted line: unstained control, Solid line: a marker of interest)

**Fig. 2:**
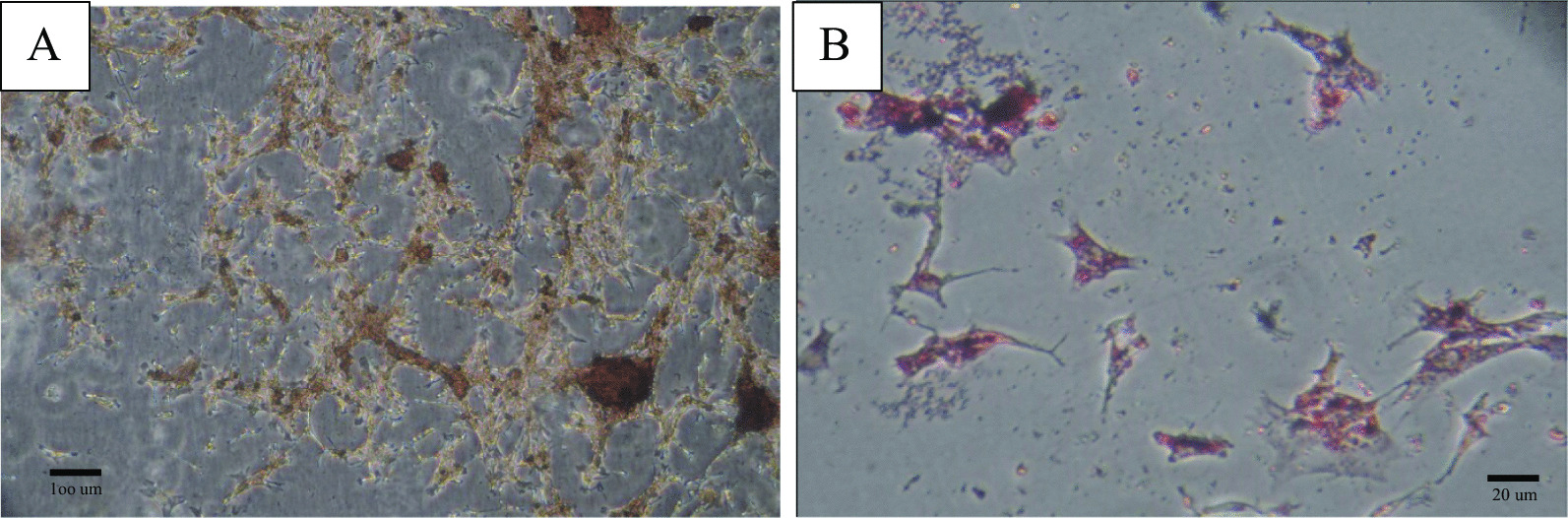
**A** Osteogenic and **B** adipogenic differentiation of hUC-MSCs (magnification ×100 (**A**) and ×400 (**B**) from inverted phase microscope). *hUC-MSCs* human umbilical cord-derived mesenchymal stromal cells

UC-MSCs were obtained from the umbilical cord tissues of healthy mothers (*n* = 10) with informed consent and prepared for injection. Briefly, the umbilical cords were washed several times with PBS to obtain clean, bloodless tissue. The blood vessels were delicately separated by a longitudinal and transverse incision of the Wharton's jelly. Then, it was cut up into 1–2 mm pieces and treated, respectively, with 0.2% Collagenase/Dispase (Boehringer Mannheim GmbH, Germany) for 1 h and then 0.125% Trypsin–EDTA (Gibco, USA) for 30 min at 37 °C with agitation. After centrifugation (1500 rpm, 5 min) and removal of the supernatant (3 times), small pieces were seeded in 25 cm^2^ flasks (SLP, South Korea) and maintained in a culture medium including minimum essential medium-α (alpha-MEM, Biowest/South America) supplemented with 20% fetal bovine serum (Biowest/South America) and 1% penicillin/streptomycin (Gibco, USA) and then incubated in a humidified incubator at 37 °C under 5% CO2. After observing the several colony-forming units (CFUs), the tissues were removed from the culture flasks. The fibroblast-like adherent cells were digested and re-plated for expansion to passage 3 [27]. Then, UC-MSCs were characterized based on the International Society for Cellular Therapy (ISCT) Guidelines by flow cytometry analysis (Fig. 1) and differentiation ability into osteocytes (Fig. 2A) and adipocytes (Fig. 2B) [27]. Briefly, osteogenic differentiation of MSCs was assessed with Alizarin Red S (Kia zist, Iran) staining. In addition, Oil Red O (Kia zist, Iran) staining was used to examine the capacity of MSCs to differentiate along an adipogenic lineage.

To ensure sterility and lack of bacterial, yeast, fungal and mycoplasma contamination, endotoxin, Bactec and PCR tests were performed on the cell samples before each injection. The cell viability percentage was between 95 and 98% before each infusion using Trypan Blue staining method. Finally, after reaching 80% confluence, the UC-MSCs were detached using 0.125% trypsin–EDTA (Gibco, USA) and were suspended in 100 ml of normal saline for each infusion. All the procedures mentioned above were performed in a Grade B cleanroom with GMP requirements.

### End points

The primary outcome of this study is to evaluate the safety of three doses injection of UC-MSCs in COVID-19-induced ARDS patients. Also, as the secondary end point, it is an exploratory pilot study to determine possible changes in specific biomarkers of inflammatory dysregulation and their alteration following MSCs therapy in these patients.

### Trial participants, enrollment and follow-up period

In this trial, 20 pneumonia COVID-19 patients confirmed by RT-PCR or CT scan image with non-severe ARDS (120 ≤ SPO_2_/FIO_2_ ≤ 315) [28] were considered eligible and by clinicians (emergency medicine physician or intensivist) divided into two groups, including a standard care group (control group) and standard care plus umbilical cord mesenchymal stem cell infusion group (intervention group). In this study, the standard care was in accordance with the general treatment protocols approved in that time period and usually included complementary therapies, dexamethasone and remdesivir. Antibiotics and other necessary treatments were also prescribed according to the patient's condition and the physicians' discretion.

The results of PCR tests were reviewed and confirmed by clinicians, and the CT scans' results were confirmed by radiologists. All patients had a positive PCR-test and were admitted to the ICU during the study. Although patients could not tell us the exact time of disease onset and were usually delayed in performing PCR testing, at least one week had passed since the onset of symptoms in all patients. Besides, all patients or their representatives were informed of the details of the study by clinicians and entered the study after filling out an informed written consent form. Follow-up period was 17 days.

The inclusion and exclusion criteria were:

#### Inclusion criteria

- Male or female in the age range of 18 to 75 years.
- Signing an informed written consent form.
- Confirmed COVID-19 patients with non-severe ARDS (120 ≤ SPO_2_/FIO_2_ ≤ 315).
- Required supplemental oxygen.

#### Exclusion criteria

- Severe underlying diseases (like auto-immune disease, cancer, heart, liver or kidney dysfunction).
- Pregnancy or breastfeeding.
- Failure to receive a full three doses of injections.
- Evidence of severe side effects after cell transplantation.
- Concurrent enrollment in other studies.
- Co-infection with other viruses (like HIV, HBS, HBV, influenza virus, etc.).

### Procedures

Intravenously (from peripheral vein) three injections of UC-MSCs (1 × ${10}^{6}$ cells/kg BW per injection) every other day (days 1, 3 and 5) were performed for patients. The infusion time was approximately 30–45 min (approximately 50 drops/min). In a placebo-control group, 100 ml of normal saline was injected. In all stages of the study, patients got standard care based on their particular situations.

### Data collection

This trial was conducted at Imam Reza Hospital, Mashhad University of Medical Sciences, Mashhad, Iran, and lasted from July 2020 to May 2021. All clinical and paraclinical data of patients at this time were extracted and recorded from the patients' records by the research team.

### Data safety monitoring

#### Assessment of clinical and paraclinical parameters and follow-up period

The main aim of the present study was to investigate the safety of 3 doses of UC-MSCs transplantation with an interval of one day in COVID-19-induced ARDS patients. For this purpose, to check safety, patients were closely monitored by a clinician for 24 to 48 h after each injection for any possible allergic reactions from any skin lesion to sever anaphylactic reaction. If any of the severe symptoms were observed, the patient was immediately excluded from the study.

### Biological assays

To explore the UC-MSCs effects, patients' respiratory parameters, including the Spo2/Fio2 ratio and patients’ pre- and post-lung status, were assessed. Changes in the serum CRP levels and level of inflammatory and proinflammatory cytokines during the treatment period were also examined (ELISA, Karmania Pars gene, Iran). All relevant factors were measured four times on 0, 2, 7 and 14 days after the second injection (days 0, 5, 10 and 17). However, the results of CT scan images were evaluated in two stages (before the intervention and two weeks after the intervention).

### Statistical analysis

Shapiro–Wilk test was applied to test the normality distribution. Generalized linear model (GLM) and repeated measures ANOVA (RM ANOVA) analysis were used to test the effect of time on normally distributed data. Non-normally distributed continuous variables are reported as the median (interquartile range, IQR). The comparison of the medians between two related groups was made by paired *T* test or Wilcoxon signed ranks. Continuous variables are expressed as the mean (for normally distributed data) or median (for non-normal distributed data) ± SEM (standard error of the mean/median). The data were analyzed using the statistical package IBM SPSS version 23.0, and the significance level was regarded at *P* < 0.05.

## Results

### Patients' clinical characterization and safety results

In this study, 10 patients participated in the control group (4 female and 6 male) with a mean 61.3 ± 5.34 age. Also, in the intervention group, 10 patients (3 female and 7 male) with a mean age of 62.00 ± 2.42 were considered eligible and entered the study. However, during the study period, in the intervention group one of the patients due to dissatisfaction withdrew from the study after receiving the first dose of MSCs and was excluded from the study (based on the initial agreement of exclusion criteria) and replaced with another patient. This patient was evaluated for safety and outcome and then underwent routine treatments. During the follow-up period, all patients were observed with the Hemovigilance protocol. The obtained results were promising and showed that MSCs transplantation was safe with no major adverse effect in COVID-19-induced ARDS patients. The mild headache was the most common side effect during the injections, which was eliminated quickly with complementary therapies. However, on the 13th day after the onset of the disease, one death was reported in the control group, and in the intervention group, two patients died on the 6th and 17th days, respectively, but examinations by specialist physicians showed no association between death and MSCs injections. The cause of death in these patients in intervention group was the progression of ARDS from moderate to severe, which was determined by examining the respiratory and clinical indices by clinicians. Also, in the control group, pulmonary embolism was diagnosed as the cause of death. Table 1 summarizes the patient's demographic data and clinical characterization of them at baseline.

**Table 1:** Demographic data and clinical characterization of patients enrolled in clinical trial

Group	Patient number	Age range	Sex	Co-morbidities	RT-PCR at baseline	Adverse events	Clinical outcome
Control group	P1	60–69	2	Hypertension	+	NO	Died
	P2	60–69	2	Diabetes	+	NO	Discharge
	P3	60–69	1	NO	+	NO	Discharge
	P4	60–69	2	NO	+	NO	Discharge
	P5	50–59	2	Hypertension	+	NO	Discharge
	P6	50–59	1	NO	+	NO	Discharge
	P7	60–69	1	NO	+	NO	Discharge
	P8	50–59	2	NO	+	NO	Discharge
	P9	60–69	2	NO	+	NO	Discharge
	P10	60–69	1	Hypertension	+	NO	Discharge
Intervention group	P1	50–59	1	Diabetes	+	Mild headache	Died
	P2	60–69	2	NO	+	Mild headache	Discharge
	P3	50–59	1	NO	+	NO	Discharge
	P4	60–69	2	Hypertension	+	NO	Died
	P5	70–79	2	NO	+	NO	Discharge
	P6	60–69	2	Diabetes	+	NO	Discharge
	P7	60–69	2	NO	+	Mild headache	Discharge
	P8	60–69	1	Mild anemia	+	NO	Discharge
	P9	60–69	2	NO	+	Mild fever	Discharge
	P10	50–59	2	NO	+	NO	Discharge

(In gender or sex column, 1 = female and 2 = male)

In this study, the standard care was in accordance with the general treatment protocols approved in that time period and usually included complementary therapies, dexamethasone and remdesivir. Antibiotics and other necessary treatments were also prescribed according to the patient's condition and the physicians' discretion

### Oxygenation

All participants in this study were in the none-severe group in terms of ARDS index at the onset of the study based on their symptoms and the first ratio of 120 ≤ SPO_2_/FIO_2_ ≤ 315, which was in line with WHO guideline [28].

**Fig. 3:**
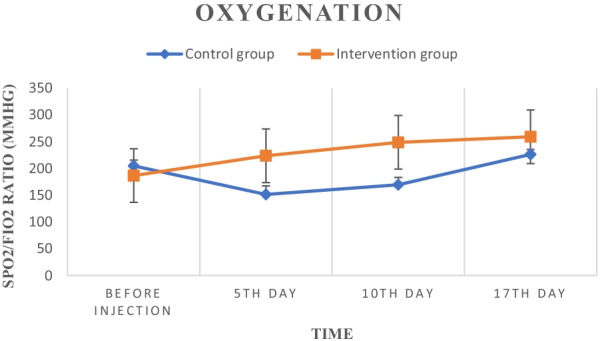
Oxygenation changes (SPO2/FIO2 ratio) in the control and intervention groups during the study

At the beginning of the study, the SPO2/FIO2 ratio was 204.81 ± 10.84 in the control group and 186.82 ± 10.61 in the intervention group, which there was no statistically significant difference between these two groups (*P* = 0.36). This ratio significantly decreased to 151.38 ± 16.05 in the control group, while that increased to 223.83 ± 17.38 significantly in the intervention group on the 5th day. Also, a significant difference was observed between these two groups in this time (*P* = 0.003). Changes in the SPO2/FIO2 ratio in the control group on the 10th day (169.49 ± 14.08) again were significantly decreased compared to the first day (*P* = 0.045), while those were significantly (*P* = 0.029) increased in the intervention group (248.88 ± 24.3) compared to the first day. Finally, on the 17th day, a non-significant increase (*P* = 0.055) was observed in the control group (226.24 ± 9.43) compared to the first day of the study, while in the intervention group (259.28 ± 24.62), a significant increase (*P* = 0.018) was observed at the same time. Figure 3 shows the mentioned changes.

### Serum CRP levels

**Fig. 4:**
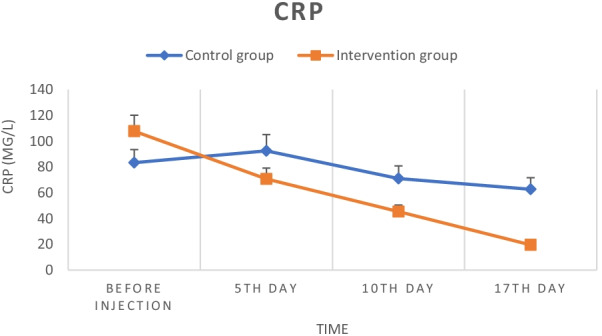
Serum CRP levels in the control and intervention groups during the study

At the study onset, the serum CRP levels in the two groups of control (83.36 ± 9.4) and intervention (107.73 ± 7.2) showed a significant difference (*P* = 0.04) which after five days compared to day zero showed a significant increase in the control group (92.55 ± 8.66, *P* = 0.008) and significant decrease in the intervention group (70.68 ± 5.82, *P* = 0.005). Also on the tenth day, a significant decrease was observed in both control (71.04 ± 6.13, *P* = 0.01) and intervention (45.37 ± 3.95, *P* = 0.008) groups. On the seventeenth day, this rate showed a significant decrease in both control (62.66 ± 6.77, *P* = 0.008) and intervention (19.55 ± 0.89, *P* = 0.005) groups. After the intervention, a significant difference (*P* < 0.05) was observed between the two groups at all times and the decrease in CRP levels was greater in the intervention group than in the control group (Fig. 4).

### Lung imaging

**Fig. 5:**
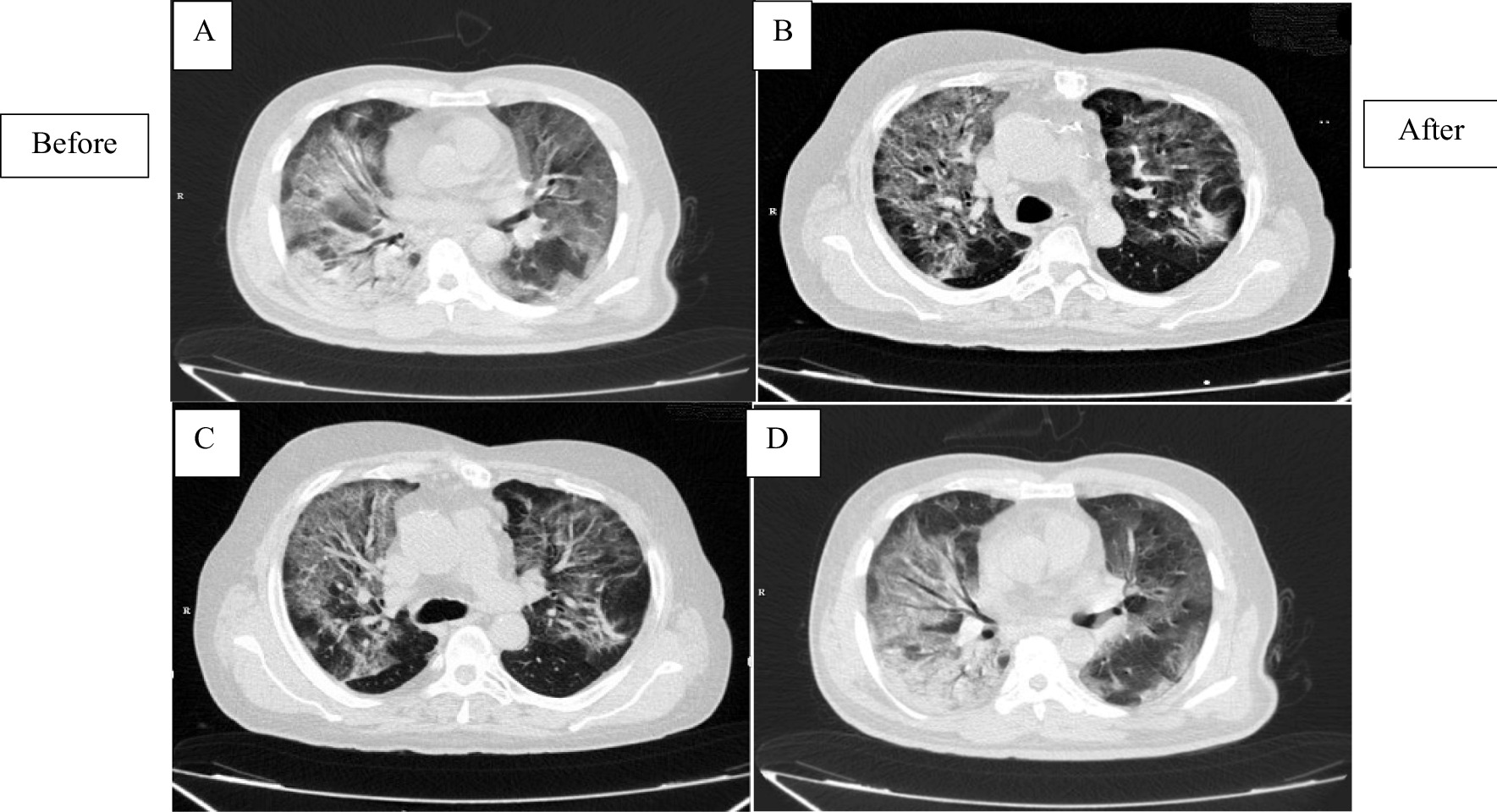
Schematic before and after lung CT scan of patients. **A** and **B** are representative images of a patient in intervention group (before and after treatment)/**C** and **D** are representative images of a patient in control group (before and after study)

Based on the score determined by the radiologists, the CT scan results of the patients showed insignificant changes in the short time. Since the same result was observed in all patients, here the before- and after-CT image of a patient from each group is shown (Fig. 5A–D).

**Cytokine analysis**

### IL-6 levels

**Fig. 6:**
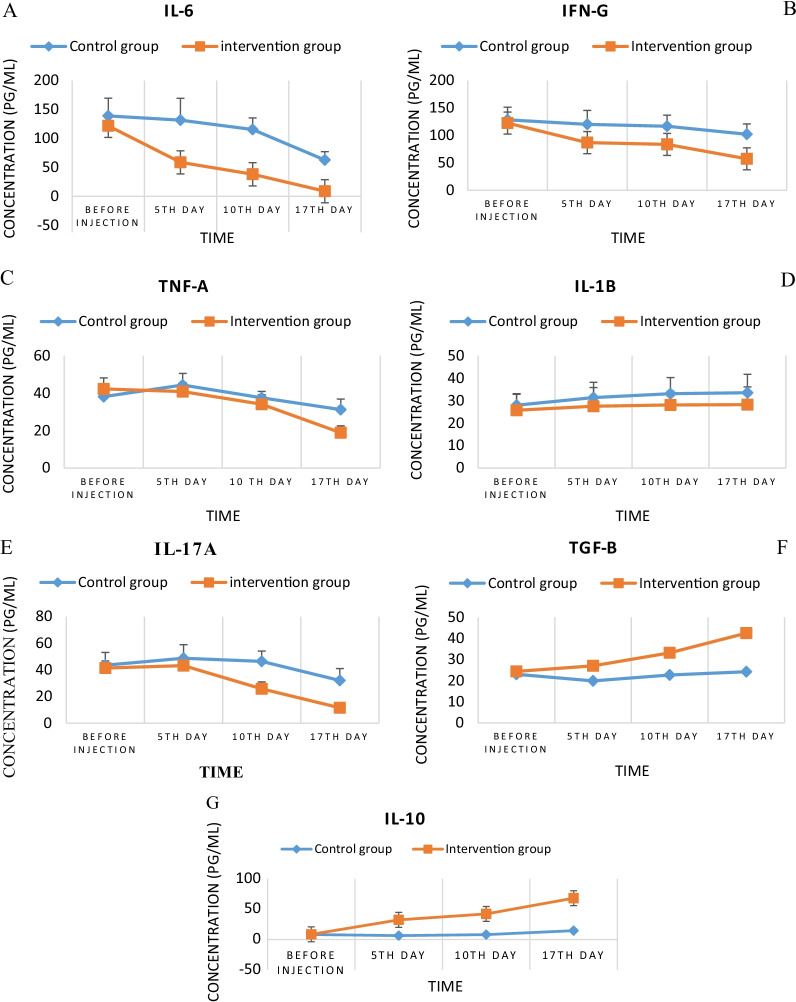
**A** Serum IL-6 levels in the control and intervention groups during the study. **B** Serum IFN-g levels in the control and intervention groups during the study. **C** Serum TNF-α levels in the control and intervention groups during the study. **D** Serum IL-1β levels in the control and intervention groups during the study. **E** Serum IL-17A levels in the control and intervention groups during the study. **F** Serum TGF-β levels in the control and intervention groups during the study. **G** Serum IL-10 levels in the control and intervention groups during the study. All measurements were performed by ELISA method

The initial measurement revealed that the serum level of cytokine IL-6 in the control group was (138.906 ± 30.68), which compared to the intervention group (121.65 ± 25.78) statistically (*P* < 0.05) was no significant difference at the onset of the study. Treatment with UC-MSCs in the intervention group significantly reduced this level on the fifth (58.70 ± 12.18, *P* = 0.008), tenth (38.03 ± 10.3, *P* < 0.001) and seventeenth (8.78 ± 1.12, *P* < 0.0001) days, respectively. Also, in the control group, a decreasing trend in the level of this cytokine was observed during the study period. This decrease on the fifth day (131.63 ± 37.56) compared to the first day was not statistically significant, but on the tenth (115.28 ± 20.13, *P* = 0.035) and seventeenth days (62.78 ± 14.2, *P* < 0.001) it was significant. Also, on the tenth and seventeenth days, a significant difference (*P* < 0.05) was observed between the two intervention and control groups and the rate of reduction of this inflammatory cytokine in the intervention group was significantly higher than the control group (Fig. 6A).

### IFN-g levels

During the study, the level of inflammatory cytokine IFN-g in both control and intervention groups in the patient's serum showed a decreasing trend. This amount was 128.37 ± 23.11 in the control group and 122.53 ± 27.11 in the intervention group at the study onset, with a statistically non-significant difference. Stem cells treatment resulted in a significant reduction in this cytokine on the 5th (86.92 ± 15.12, *P* < 0.0001), 10th (83.66 ± 17.14, *P* < 0.0001) and 17th days (57.37 ± 8.9, *P* < 0.0001) compared to before injection. Also, in the control group on the 5th day, there was a non-significant decrease (120.14 ± 25.47), while on the 10th (116.8 ± 20.12, *P* < 0.001) and 17th (102.13 ± 18.94, *P* < 0.001) days, a significant decrease compared to the onset of study was observed. Besides, on the fifth (*P* = 0.002), 10th (*P* = 0.002) and seventeenth (*P* < 0.0001) days, a significant decrease was observed in the intervention group compared to the control group, respectively, which indicates a greater decrease in this cytokine in the intervention group than the control group (Fig. 6B).

### TNF-α levels

The initial serum level of cytokine TNF-α in the two groups of control (38.11 ± 4.17) and intervention (42.33 ± 5.9) showed a non-significant difference. Simultaneously with the disease progression on the fifth day of the study, this cytokine level in the control group increased significantly (44.30 ± 6.21, *P* = 0.001), but in the intervention group, it decreased non-significantly (40.81 ± 4.8, *P* = 0.7) compared to the onset of the study. On the 10th day, a non-significant decrease (37.52 ± 3.4, *P* = 0.54) in the control group and a significant decrease in the intervention group (34.05 ± 5.1, *P* < 0.0001) was observed. After 17 days, a significant decrease was observed in both control (31.20 ± 5.6, *P* < 0.0001) and intervention groups (18.88 ± 3.7, *P* < 0.0001). Also, on the 17th day, a significant difference (*P* < 0.0001) was observed between the intervention and control groups (Fig. 6C).

### IL-1B levels

At the study'-onset, there was no significant difference in cytokine IL-1B levels between the control (27.96 ± 5.11) and intervention groups (25.70 ± 7.15). The level of this cytokine on the 5th day of the study increased significantly in control (31.37 ± 6.78, *P* < 0.001) and intervention group (27.49 ± 8.34, *P* < 0.001). Also, a significant increase was observed on the 10th day in both control (33.08 ± 7.14) and intervention groups (28.07 ± 6.12). In addition, on the 17th day, a significant increasing trend was observed in both control (33.53 ± 8.16) and intervention groups (28.20 ± 7.89). On the other hand, in all three time periods of the 5th, tenth and 17th days, there was a significant difference (*P* < 0.05) between the two control and intervention groups and the decreasing trend was more in the intervention group (Fig. 6D).

### IL-17 A levels

The initial level of inflammatory cytokine IL-17 A in the two groups of control (43.52 ± 6.41) and intervention (41.42 ± 5.32) showed no statistically significant difference. On the 5th day, a non-significant increase was observed in both control (48.75 ± 7.64, *P* = 0.17) and intervention groups (43.18 ± 5.11, *P* = 0.9) compared to the first day of the study. Also, on the 10th day of the disease, the level of this cytokine in the control group showed a non-significant decrease (46.37 ± 8.12, *P* = 0.4), while in the intervention group that showed a significant decrease (25.76 ± 5.18, *P* < 0.001). On the 17th day of the disease, a significant decrease in the amount of this cytokine was observed in both control (32.08 ± 4.1, *P* = 0.016) and intervention groups (11.51 ± 1.3, *P* < 0.001) (Fig. 6E).

### TGF-B levels

TGF-B cytokine levels at the beginning of the study were (23.06 ± 5.12) in control and (24.38 ± 6.15) in the intervention group, which did not show a statistically significant difference (*P* = 0.26). On the 5th day of the disease, the cytokine level in the control group significantly decreased (19.89 ± 4.76, *P* = 0.02), while in the intervention group that significantly increased (27.03 ± 7.24, *P* = 0.02). Also, on the 10th day compared to the first day of study, its level decreased insignificantly in the control group (22.70 ± 6.17, *P* = 0.18) and increased significantly in the intervention group (33.20 ± 8.12, *P* = 0.003). On the 17th day, an increasing trend was observed in the levels of this cytokine which this increase was statistically insignificant in the control group (24.24 ± 7.34) but was significant in the intervention group (42.52 ± 9.11, *P* = 0.005). Also, on the 5th, 10th and 17th days, a significant difference was observed between the two intervention and control groups and the amount of this cytokine in the intervention group compared to the control group showed a significant increase (*P* < 0.05) (Fig. 6F).

### IL-10 levels

Cytokine IL-10 at the beginning of the study showed an insignificant difference in both controls (8.19 ± 1.66) and intervention groups (8.57 ± 1.53). After 5 days of the disease, its rate significantly decreased in the control group (6.38 ± 1.23, *P* = 0.004), while that significantly increased in the intervention group (32.31 ± 6.12, *P* = 0.009). On the 10th day of the disease, a non-significant decrease was observed in the control group (8.06 ± 2.41, *P* = 0.48) and a significant increase in the level of this cytokine in the intervention group (42.13 ± 11.32, *P* < 0.0001). Besides, on the 17th day of the study, compared to the first day, a significant increase was observed in both control (14.53 ± 2.8, *P* = 0.002) and intervention groups (68.06 ± 15.12, *P* < 0.0001). A significant increase (*P* < 0.05) in the amount of this cytokine was observed in the intervention group on the 5th, 10th and 17th days compared to the control group (Fig. 6G).

## Discussion

The present study aimed to assess the safety and potency of 3 doses of UC-MSCs in COVID-19 -induced ARDS patients. All participants were non-severe in terms of ARDS index at the onset of the study, which was calculated based on their first ratio of SPO2/FIO2, and were admitted to the ICU due to moderate–severe conditions for COVID-19 disease. The obtained results were promising and showed that MSCs transplantation was safe with no major adverse effect in COVID-19-induced ARDS patients. The mild headache was the most common side effect during the injections, which was eliminated quickly with complementary therapies. In addition, improvement in respiratory function, which is evident by increasing the SPO2/FIO2 ratio after the first injection (Fig. 1), confirmed the potency of this method.

To date, many preclinical [29, 30] and clinical studies have been performed using MSCs therapy for ARDS. For example, in a phase 1, multicenter clinical trial by Wilson et al. on 9 ARDS patients, injection of 3 courses of MSCs with different doses (low to high) had no injection-related side effects [17]. Also, the obtained results from Matthay et al. study were promising. In this multicenter phase 2 clinical trial, IV transplantation of single dose of bone marrow-derived MSCs (10 × 10^6^ cells/kg PBW) on 40 moderate–severe ARDS patients was safe and well tolerated by patients. Also, 28-day and 60-day follow-up of patients in the intervention and control groups did not show any significant change in terms of mortality rate between them [19].

In the case of COVID-19 patients with pneumonia or ARDS, several clinical trials have also been registered in www.clinicaltrial.gov so far, but only a few of them have been completed and published their results [22, 31]. For example and in the same direction, 7 COVID-19 patients who did not get beneficial results from common standard care were included in a pilot study performed by Leng et al. and received 1 × ${10}^{6}$ MSCs/kg BW. According to our results, no serious side effects were observed during a 14-day follow-up period after injection and disease symptoms greatly improved [7]. However, the mortality rate in the intervention group was 20% (2 out of 10 cases), which was more than the control group with a 10% mortality rate (1 out of 10 cases), but no significant relationship was observed between cell injections and patients' death. Differences in patients' initial conditions and discrepancy in response to standard treatments can be reasons for differences in mortality rates in these two groups. On the other hand, the mortality rate of patients in this study was less than the study of Hashemian et al. with 45% mortality [22] and was relatively close to the study of Sánchez-Guijo et al. with 15% mortality rate [32]. Also, the results of patients' CT scan in this study (Fig. 3) did not show significant changes during two weeks which was expected due to the short time interval.

We also, in the present study, examined the changes of specific inflammatory and anti-inflammatory cytokines to investigate the “cytokine storm” in COVID-19 patients. It has been documented that “cytokine storm” phenomenon is directly correlated with high morbidity and mortality rate in COVID-19 patients diagnosed with ARDS [33, 34]. Cytokine storm is a hostile inflammatory response, characterized by the discharge of a wide stream of accumulated inflammatory cytokines like interleukin-6 (IL-6), interleukin-1B (IL-1B), IL-8, tumor necrosis factor-alpha (TNF-α) and interferon-gamma (IFN-g) which could enhance the SARS-COV-2 invasion by sending alarming signals to the immune cells and recall them to the inflamed sites [35]. Following this aggressive stimulation, immune cells discharge lots of free radicals, which coincides with the disease progression leading to ARDS and multi-organ failure in COVID-19 patients [36, 37]. Besides, it is approved that cytokine storms severity is directly associated with the severity of clinical symptoms of COVID-19 patients [38].

As our results show, the patients' inflammatory cytokines at the study onset were relatively high. MSCs injection significantly reduced the level of most of these cytokines (IL-6 (Fig. 6A), IFN-g (Fig. 6B), TNF-α (Fig. 6C), IL-17 A (Fig. 6E), except cytokine IL-1B (Fig. 6D)) while increasing the serum level of anti-inflammatory cytokines including TGF-B (Fig. 6F) and IL-10 (Fig. 6G), which these results were largely consistent with similar studies. For example, Shu et al. in their clinical trial, performed on 41 COVID-19 patients which 12 of whom received 2 × ${10}^{6}$ UC-MSCs, for one time, found that cells could potentially reduce the serum IL-6 and CRP levels and improve CT scan images over a 28-day follow-up period [39]. Similarly, in the present study, a significant decrease in serum CRP levels was observed following cell injection (Fig. 2). In addition, another case report study reported that two times intravenous administration of UC-MSCs with convalescent plasma significantly improved the pulmonary microenvironment and recovered pulmonary damage caused by inflammation via cytokine storm inhibition [40]. Also, in agreement with our results, obtained results from Lanzoni et al. study revealed that intravenous transplantation of UC-MSCs in two different doses could significantly decrease the inflammatory cytokine (TNF-α) and increase the anti-inflammatory cytokine (IL-10) in COVID-19-induced ARDS patients [21].

Also, in our study, we observed a dramatic elevation in serum cytokine IL-6 and IL-17 levels in patients before the onset of the study. It has been proven that IL-6 triggers the activation of TH17 cells in the interaction between T cell-dendritic cells [41]. In this regard, there is a hypothesis that elevated levels of IL-6 following the viruses' entry into the immune system may be one of the reasons for elevated TH17 cells in COVID-19 patients [42]. Several clinical trials have reported a significant decrease in serum IL-6 levels after MSCs transplantation [39, 43] consistent with our results.

As mentioned above, so far, various studies have shown the potency of MSCs to decrease cytokine storm in COVID-19-induced ARDS patients [22, 44]. Although the main underlying mechanism in this process is not yet clear, there are several hypotheses in this regard. For instance, a recent study shows that UC-MSCs and their derivatives can potentially suppress monocyte activation and IL-6 production and subsequently inhibit the cytokine storm and improve the clinical condition of patients [45]. In addition, MSCs have strong anti-inflammatory properties through paracrine secretion of various anti-inflammatory cytokines such as IL-10, TGF-B, IL-4 and prostaglandin E2 (PGE2) which can modulate the overactive immune system [46, 47]. In combat to virus entry into the body, MSCs also secret many soluble factors like TGF-B, PGE2, nitric oxide (NO) and indoleamine 2,3-dioxygenase (IDO), which modulate the immune system via two ways [48, 49]. Firstly, inhibiting the activation and expansion of T-helper 1 (TH1) and TH17 cells reduces the inflammatory cytokines IFN-g and interleukin-17 [50]. Secondly, by suppressing dendritic cell (DC) activation, inhibiting their formation from monocytes and reducing IL-12 production, MSCs can suppress DCs as the central antigen presenting cells (APCs) [50]. Moreover, the documents show that MSCs by homing in the lung through intravenous administration could potentially boost lung function via avoiding pulmonary fibrosis, deserving lung pneumocytes and enhancing the pulmonary microenvironment [4, 7, 44]. Another protective mechanism of MSCs on lungs is secreting the keratinocyte growth factor which leads to decline in alveolar edema and endothelial permeability [51].

Our study had limitations and advantages over similar clinical trials. One of the limitations of our study was the short follow-up period of patients that since our study began in the first peaks of the pandemic and the peak workload of doctors and hospitals, there was no possibility of longer follow-up of patients. However, shorter follow-up periods have also been reported [7, 44, 52]. In addition, the study's small sample size due to the newness of the disease and financial issues was another limitation of this study which we suggest to be done with a larger population in future trials. One of the key advantages of this study was using an umbilical cord source for stem cell injections which is a safe and noninvasive method with more MSCs density compared to extracting cells from adipose tissue or bone marrow. Also, the use of fresh cells compared to cryopreserved cells acted as a double-edge sword in this study. On the one hand, the use of fresh cells limits the time to recruit patients, and we were only able to select eligible patients when the cells were close to the third passage. On the other hand, the use of these cells decreases the risk of allergic reactions in patients due to the lack of DMSO and increases the percentage of cell survival. Besides, having a control group for better data comparison was another advantage of this study.

## Conclusion

In conclusion, our results indicated that MSC therapy in COVID-19-induced ARDS patients could down-regulate the disease progression safely due to improvement in respiratory function and cytokine storm reduction. Our results also showed that increasing the number of injections (more than once) can lead to an increase in patients' recovery. Although our study results are promising, further clinical investigations with larger sample size and more extended follow-up periods are required to confirm these results.

## Data Availability

All data generated or analyzed during this study are included in this published article.

## Abbreviations

ARDS: Acute respiratory distress syndrome
COVID-19: Coronavirus disease 2019
MSCs: Mesenchymal stromal cells
UC-MSCs: Umbilical cord-derived MSCs
CRP: C reactive protein
SPO2: Oxygen saturation
FIO2: The fraction of inspired oxygen
TNF-α: Tumor necrosis factor-alpha
CT: Computed tomography
SARS-CoV-2: Severe acute respiratory syndrome coronavirus 2
ACE2: Angiotensin-converting enzyme 2
ICUs: Intensive care units
SLE: Systemic lupus erythematosus
ALS: Amyotrophic lateral sclerosis
GVHD: Graft-versus-host disease
WHO: World Health Organization
ISCT: International Society for Cellular Therapy
GMP: Good manufacturing practice
IFN-g: Interferon-gamma
TGF-B: Tumor growth factor-B
IDO: Indoleamine 2,3-dioxygenase
DMSO: Dimethyl sulfoxide

## References

[1] Li Q, Guan X, Wu P, Wang X, Zhou L, Tong Y (2020). Early transmission dynamics in Wuhan, China, of novel coronavirus-infected pneumonia. New Engl J Med..

[2] Xu X-W, Wu X-X, Jiang X-G, Xu K-J, Ying L-J, Ma C-L (2020). Clinical findings in a group of patients infected with the 2019 novel coronavirus (SARS-Cov-2) outside of Wuhan, China: retrospective case series. BMJ.

[3] Holshue ML, DeBolt C, Lindquist S, Lofy KH, Wiesman J, Bruce H (2020). First case of 2019 novel coronavirus in the United States. New Engl J Med..

[4] Farkhad NK, Reihani H, Moghadam AA, Moghadam AB, Tavakol-Afshari J (2021). Are mesenchymal stem cells able to manage cytokine storm in COVID-19 patients? A review of recent studies. Regen Ther..

[5] Fang Y, Liu H, Huang H, Li H, Saqi A, Qiang L (2020). Distinct stem/progenitor cells proliferate to regenerate the trachea, intrapulmonary airways and alveoli in COVID-19 patients. Cell Res.

[6] Zhou P, Yang X-L, Wang X-G, Hu B, Zhang L, Zhang W (2020). A pneumonia outbreak associated with a new coronavirus of probable bat origin. Nature.

[7] Leng Z, Zhu R, Hou W, Feng Y, Yang Y, Han Q (2020). Transplantation of ACE2-mesenchymal stem cells improves the outcome of patients with COVID-19 pneumonia. Aging Dis.

[8] Magro G (2020). SARS-CoV-2 and COVID-19: is interleukin-6 (IL-6) the ‘culprit lesion’ of ARDS onset? What is there besides Tocilizumab? SGP130Fc. Cytokine: X.

[9] Kao K-C, Hu H-C, Chang C-H, Hung C-Y, Chiu L-C, Li S-H (2015). Diffuse alveolar damage associated mortality in selected acute respiratory distress syndrome patients with open lung biopsy. Crit Care.

[10] Huang C, Wang Y, Li X, Ren L, Zhao J, Hu Y (2020). Clinical features of patients infected with 2019 novel coronavirus in Wuhan, China. Lancet.

[11] Zhou F, Yu T, Du R, Fan G, Liu Y, Liu Z (2020). Clinical course and risk factors for mortality of adult inpatients with COVID-19 in Wuhan, China: a retrospective cohort study. Lancet.

[12] Neri S (2019). Genetic stability of mesenchymal stromal cells for regenerative medicine applications: a fundamental biosafety aspect. Int J Mol Sci.

[13] Farkhad NK, Mahmoudi A, Mahdipour E (2021). How similar are human mesenchymal stem cells derived from different origins? A review of comparative studies. Curr Stem Cell Res Ther.

[14] Manchikanti L, Centeno CJ, Atluri S, Albers SL, Shapiro S, Malanga GA (2020). Bone marrow concentrate (BMC) therapy in musculoskeletal disorders: evidence-based policy position statement of American Society of Interventional Pain Physicians (ASIPP). Pain Phys.

[15] Tavakol-Afshari J, Boroumand AR, Farkhad NK, Moghadam AA, Sahab-Negah S, Gorji A (2021). Safety and efficacy of bone marrow derived-mesenchymal stem cells transplantation in patients with amyotrophic lateral sclerosis. Regen Ther.

[16] Wu Y, Cao Y, Li X, Xu L, Wang Z, Liu P (2014). Cotransplantation of haploidentical hematopoietic and umbilical cord mesenchymal stem cells for severe aplastic anemia: successful engraftment and mild GVHD. Stem Cell Res.

[17] Wilson JG, Liu KD, Zhuo H, Caballero L, McMillan M, Fang X (2015). Mesenchymal stem (stromal) cells for treatment of ARDS: a phase 1 clinical trial. Lancet Respir Med.

[18] Liu KD, Wilson JG, Zhuo H, Caballero L, McMillan ML, Fang X (2014). Design and implementation of the START (STem cells for ARDS Treatment) trial, a phase 1/2 trial of human mesenchymal stem/stromal cells for the treatment of moderate–severe acute respiratory distress syndrome. Ann Intensive Care.

[19] Matthay MA, Calfee CS, Zhuo H, Thompson BT, Wilson JG, Levitt JE (2019). Treatment with allogeneic mesenchymal stromal cells for moderate to severe acute respiratory distress syndrome (START study): a randomised phase 2a safety trial. Lancet Respir Med.

[20] Simonson OE, Mougiakakos D, Heldring N, Bassi G, Johansson HJ, Dalén M (2015). In vivo effects of mesenchymal stromal cells in two patients with severe acute respiratory distress syndrome. Stem Cells Transl Med.

[21] Lanzoni G, Linetsky E, Correa D, Alvarez R, Marttos A, Hirani K (2020). Umbilical cord-derived mesenchymal stem cells for COVID-19 patients with acute respiratory distress syndrome (ARDS). CellR4 Repair Replacement Regener Reprogram.

[22] Hashemian S-MR, Aliannejad R, Zarrabi M, Soleimani M, Vosough M, Hosseini S-E (2021). Mesenchymal stem cells derived from perinatal tissues for treatment of critically ill COVID-19-induced ARDS patients: a case series. Stem Cell Res Ther.

[23] Lalu MM, McIntyre L, Pugliese C, Fergusson D, Winston BW, Marshall JC (2012). Safe ty of cell therapy with mesenchymal stromal cells (safecell): a systematic review and meta-analysis of clinical trials. PLoS ONE.

[24] Orleans L, is Vice H, Manchikanti L (2020). Expanded umbilical cord mesenchymal stem cells (UC-MSCs) as a therapeutic strategy in managing critically ill COVID-19 patients: the case for compassionate use. Pain Phys.

[25] Wysoczynki M, Khan A, Bolli R (2018). New paradigms in cell therapy: repeated dosing, intravenous delivery, immunomodulatory actions, and new cell types. Circ Res.

[26] Qin H, Zhao A (2020). Mesenchymal stem cell therapy for acute respiratory distress syndrome: from basic to clinics. Protein Cell.

[27] Seshareddy K, Troyer D, Weiss ML (2008). Method to isolate mesenchymal-like cells from Wharton's Jelly of umbilical cord. Methods Cell Biol.

[28] Organization WH. Clinical management of severe acute respiratory infection (SARI) when COVID-19 disease is suspected: interim guidance, 13 March 2020. World Health Organization; 2020.

[29] Curley GF, Ansari B, Hayes M, Devaney J, Masterson C, Ryan A (2013). Effects of intratracheal mesenchymal stromal cell therapy during recovery and resolution after ventilator-induced lung injury. Anesthesiology.

[30] Curley GF, Hayes M, Ansari B, Shaw G, Ryan A, Barry F (2012). Mesenchymal stem cells enhance recovery and repair following ventilator-induced lung injury in the rat. Thorax.

[31] Chen H, Zhang L, He Z, Wang D, Liu L, Zhang W (2020). Systemic administration of human umbilical cord-derived mesenchymal stem cells effectively ameliorates the outcomes of a critically ill elderly patient with COVID-19 with multiple comorbidities: a case report. World Acad Sci J..

[32] Sánchez-Guijo F, García-Arranz M, López-Parra M, Monedero P, Mata-Martínez C, Santos A (2020). Adipose-derived mesenchymal stromal cells for the treatment of patients with severe SARS-CoV-2 pneumonia requiring mechanical ventilation. A proof of concept study. EClinicalMedicine.

[33] Ruan Q, Yang K, Wang W, Jiang L, Song J (2020). Clinical predictors of mortality due to COVID-19 based on an analysis of data of 150 patients from Wuhan, China. Intensive Care Med.

[34] Miao Y, Fan L, Li J-Y (2020). Potential treatments for COVID-19 related cytokine storm-beyond corticosteroids. Front Immunol.

[35] Tisoncik JR, Korth MJ, Simmons CP, Farrar J, Martin TR, Katze MG (2012). Into the eye of the cytokine storm. Microbiol Mol Biol Rev.

[36] Sinha P, Matthay MA, Calfee CS (2020). Is a “cytokine storm” relevant to COVID-19?. JAMA Intern Med.

[37] Ragab D, Salah Eldin H, Taeimah M, Khattab R, Salem R (2020). The COVID-19 cytokine storm; what we know so far. Front Immunol.

[38] Liu J, Li S, Liu J, Liang B, Wang X, Wang H (2020). Longitudinal characteristics of lymphocyte responses and cytokine profiles in the peripheral blood of SARS-CoV-2 infected patients. EBioMedicine.

[39] Shu L, Niu C, Li R, Huang T, Wang Y, Huang M (2020). Treatment of severe COVID-19 with human umbilical cord mesenchymal stem cells. Stem Cell Res Ther.

[40] Peng H, Gong T, Huang X, Sun X, Luo H, Wang W (2020). A synergistic role of convalescent plasma and mesenchymal stem cells in the treatment of severely ill COVID-19 patients: a clinical case report. Stem Cell Res Ther.

[41] Kimura A, Kishimoto T (2010). IL-6: regulator of Treg/Th17 balance. Eur J Immunol.

[42] Chi Z, Zhao W, Jia-Wen L, Hong Z, Gui-Qiang W. The Cytokine release syndrome (CRS) of severe COVID-19 and interleukin-6 receptor (IL-6R) antagonist tocilizumab man be the key to reduce the mortality. 2020. https://www.ncbi.nlm.nih.gov/pmc/articles/PMC7118634/pdf/main.pdf. 10.1016/j.ijantimicag.2020.105954 PMC7118634 32234467

[43] Shi L, Huang H, Lu X, Yan X, Jiang X, Xu R (2021). Effect of human umbilical cord-derived mesenchymal stem cells on lung damage in severe COVID-19 patients: a randomized, double-blind, placebo-controlled phase 2 trial. Signal Transduct Target Ther.

[44] Chen X, Shan Y, Wen Y, Sun J, Du H (2020). Mesenchymal stem cell therapy in severe COVID-19: a retrospective study of short-term treatment efficacy and side effects. J Infect.

[45] Shao M, Xu Q, Wu Z, Chen Y, Shu Y, Cao X (2020). Exosomes derived from human umbilical cord mesenchymal stem cells ameliorate IL-6-induced acute liver injury through miR-455-3p. Stem Cell Res Ther.

[46] Weiss ARR, Dahlke MH (2019). Immunomodulation by mesenchymal stem cells (MSCs): mechanisms of action of living, apoptotic, and dead MSCs. Front Immunol.

[47] Debuc B, Smadja DM (2021). Is COVID-19 a new hematologic disease?. Stem Cell Rev Rep.

[48] Le Blanc K, Mougiakakos D (2012). Multipotent mesenchymal stromal cells and the innate immune system. Nat Rev Immunol.

[49] Zhang W, Ge W, Li C, You S, Liao L, Han Q (2004). Effects of mesenchymal stem cells on differentiation, maturation, and function of human monocyte-derived dendritic cells. Stem Cells Dev.

[50] Jiang X-X, Zhang Y, Liu B, Zhang S-X, Wu Y, Yu X-D (2005). Human mesenchymal stem cells inhibit differentiation and function of monocyte-derived dendritic cells. Blood.

[51] Monguió-Tortajada M, Bayes-Genis A, Rosell A, Roura S (2021). Are mesenchymal stem cells and derived extracellular vesicles valuable to halt the COVID-19 inflammatory cascade? Current evidence and future perspectives. Thorax.

[52] Tao J, Nie Y, Wu H, Cheng L, Qiu Y, Fu J (2020). Umbilical cord blood-derived mesenchymal stem cells in treating a critically ill COVID-19 patient. J Infect Dev Ctries.

